# Association of the rs4646994 in *ACE* gene with susceptibility to tuberculosis in a region of the Brazilian Amazon

**DOI:** 10.1186/s41231-022-00116-6

**Published:** 2022-05-11

**Authors:** Débora C. R. F. Porchera, Diana F. V. B. Leal, Ana C. O. Braga, Pablo D. C. Pinto, Mayara N. Santana da Silva, Lucas C. Bezerra Santos, Cintia H. Braga da Silva, Giovana E. da Costa, Maria Clara da C. Barros, Aidalucy do S. C. de Athayde, Amanda de N Cohen-Paes, Cleonardo A. da Silva, Paulo P. de Assumpção, Ândrea K. C. Ribeiro-dos-Santos, Sidney E. B. dos Santos, Marianne R. Fernandes, Ney P. C. dos Santos

**Affiliations:** 1 Laboratório de Genética Humana e Médica, Universidade Federal do Pará, Rua Augusto Corrêa, N° 1, Belém, PA 66075-110 Brazil; 2 Núcleo de Pesquisa em Oncologia, Unidade de Alta Complexidade em Oncologia, Hospital Universitário João de Barros Barreto, Belém, 66073-000 Brazil

**Keywords:** Tuberculosis, Susceptibility, INDEL, Polymorphisms, *ACE*

## Abstract

**Background:**

Tuberculosis (TB) is an infectious disease caused by the bacterium *Mycobacterium tuberculosis* and represents an important global public health issue. Single-nucleotide polymorphisms and INDELs are common genetic variations that can be located in genes associated with immune response and, therefore, they may have direct implications over the phenotype of susceptibility to infections like tuberculosis. This study aimed to investigate the association between the 17 genetic polymorphisms and susceptibility to tuberculosis in a Brazilian population.

**Methods:**

This case-control study enrolled 283 individuals with active tuberculosis and 145 health care workers. Four INDELs and 13 single nucleotide polymorphisms and were genotyped using Multiplex PCR method and TaqMan SNP Genotyping Assays. Group comparisons for categorical variables were performed using the chi-squared test, whilst the t-Student test was used to analyze the continuous variables. Multiple logistic regression analyses were performed to estimate the odds ratio (OR) with 95% confidence intervals (CI). Deviation from Hardy-Weinberg equilibrium was assessed using chi-squared tests with Bonferroni correction. The results were analyzed comparing the genotypic distributions adopting the dominant model and the estimated values ​​of *p* corrected for multiple tests through FDR (False Discovery Rate) test.

**Results:**

The HWE test confirmed that the genotypic frequencies for polymorphisms were balanced. The frequency of Del allele was 73 and 75%, in cases and controls respectively. Frequency of Del allele was significantly higher in the control group than TB group. The homozygous Del/Del genotype was present in 51.6% of cases and 58.6% of controls. The rare Ins/Ins genotype was present in only 7.6% of controls and 6% of cases. The *ACE* Del/Del genotype was significantly higher in the cases than in controls revealing significant protection for TB in the domain model (OR = 0.465; *p* < 0.005).

**Conclusions:**

The Del/Del genotype of the rs4646994 in *ACE* gene was associated with susceptibility to tuberculosis. The identification of genetic variants responsible for susceptibility to tuberculosis will allow the development of new diagnostic tools for tuberculosis infection. These studies will help improve control and the future eradication of this disease.

## Introduction

Despite the BCG vaccination and chemotherapy with an effective four-drug treatment regimen, tuberculosis is the leading cause of death from a single infectious disease agent worldwide, exceeding the human immunodeficiency virus. The World Health Organization estimates that there are about 10.4 million new cases and 1.8 million deaths from TB each year. Latent tuberculosis infection affects one-third of the human population, but only 10% of infected individuals will develop active tuberculosis. Individuals with compromised immune systems have a higher risk of falling ill [1, 2].

The early reports showed that diabetes, smoking, alcohol use, drug use, and pathogen characteristics were risk factors for tuberculosis infection and active tuberculosis [3, 4]. Variations in the *Angiotensin-converting enzyme* (*ACE)* may be one among many contributing factors for tuberculosis. Interactions between the *ACE* I/D polymorphism and other risk factors can therefore influence the risk for tuberculosis disease. In the present study, we focused on the angiotensin-converting enzyme (*ACE*) Del/Del genotype that was once reported to be associated with progression of the severe acute respiratory syndrome (SARS) and COVID-19 [5, 6].

The *ACE* Ins/Del polymorphism has been one of the most studied genetic variations. The Angiotensin-converting enzyme (*ACE*) has a role in blood pressure regulation via the renin-angiotensin-aldosterone system (RAAS), in fertility, immunity, hematopoiesis, and diseases such as obesity, fibrosis, and Alzheimer’s dementia. The *ACE* is rich in microvascular endothelium and is involved in angiopathy of the lung. However, the total extent of the *ACE* influence on human health and disease is, however, still unknown [7–9].

The genotype and allele frequency of this polymorphism has been reported to be associated with several diseases including COVID-19 [10], cardiovascular disease [11], cancer [12], and diabetes mellitus and renal disease [13] in humans. In the present study, we aim to evaluate the association between *ACE* (Ins/Del) polymorphism and risk of tuberculosis in an admixture population such as the Brazilian population. These findings may be useful and provide basic information regarding our understanding of genetic predisposition and tuberculosis.

## Materials and methods

### Subjects

Blood samples were obtained from 283 Brazilian patients affected by active pulmonary tuberculosis. TB was diagnosed based on radiographic and clinical presentation, positive of acid-fast-bacilli on a sputum smear and culture for *M. tuberculosis*. A control group was composed of 145 health care workers without tuberculosis infection. Demographic and clinical characteristics of individuals were presented in Table 1. The study was approved by the Ethical Committee of João Barros Barreto Hospital, and all subjects provided written informed consent (protocol n° 350507).

**Table 1:** Demographic and clinical characteristics of individuals with tuberculosis and control group

VARIABLES	TUBERCULOSIS ***n*** = 283 (%)	CONTROL ***n*** = 145 (%)	***p VALUE***
Age (Years) ^t^	50 ± 16.84	52 ± 9.15	0.210
**Gender** ^b^			
Male	135 (47.7%)	25 (17.3%)	3.6E ^−9^
Female	148 (52.3%)	120 (82.7%)	
**Comorbidities** ^b^			
Yes	107 (35.8%)	67 (46.2%)	0.021
No	176 (64.2%)	78 (53.8%)	
**Smoking** ^b^			
Yes	77 (31.7%)	30 (20.7%)	0.002
No	206 (68.3)	115 (79.3)	
**Ancestry** ^c^			
African	0.254 ± 0.12	0.215 ± 0.11	0.0002
European	0.409 ± 0.13	0.496 ± 0.16	5.7E ^−9^
Amerindian	0.337 ± 0.13	0.289 ± 0.14	0.00004

^*t*^ Test Student T; ^b^ Fisher’s Exact Test; ^c^ Mann Whitney Test; ^c^ Mean ± Standard deviation (SD)

### DNA isolation and polymorphism genotyping

Seventeen genetic variants, INDEL, and SNP, in immune-related genes were investigated (Table 2). Genomic DNA was isolated from peripheral blood leukocytes using the phenol extraction method [14]. Four SNPs were genotyped using TaqMan® SNP Genotyping Assays (Applied Biosystems, Foster City, USA). Thirteen INDELs polymorphisms were genotyped by a single multiplex reaction with Master Mix QIAGEN® Multiplex PCR kit (Qiagen, Hilden, Germany) and the primers. Multiplex PCR products were separated and analyzed by capillary electrophoresis on the ABI 3130 Genetic Analyzer instrument (Applied Biosystems), using GS-500 LIX as a pattern of molecular weight (Applied Biosystems), G5 virtual filter matrix, and POP7 (Applied Biosystems). After data collection, samples were analyzed in GeneMapper®3.7 software (Applied Biosystems).

**Table 2:** Description of polymorphisms analyzed

GENE	IDENTIFICATION	TYPE	PRIMERS/PROBE	AMPLIFIED
***ACE2***	rs4646994	INDEL	F-5’ATCCTGTAAGCCACTGCTGGA3’ R- 5’GGCGAAACCACATAAAAGTGA3’	94–382 pb
***CCR5***	rs333	INDEL	F-5’CTCCCAGGAATCATCTTTACCA3’ R-5’TTTTTAGGATTCCCGAGTAGCA3’	146–178 pb
***SGSM03***	rs56228771	INDEL	F-5’CTAGTAGGCTCCTGGCCTCTTT3’ R-5’GCAGAACCTTGGACCTGAATAC3’	117–121 pb
***CYP2E1***	–	INDEL	F-5’TGTCCCAATACAGTCACCTCTTT3’ R-5’GGCTTTTATTTGTTTTGCATCTG3’	397–493 pb
***HLA-G***	rs371194629	INDEL	F-5’CTGTTTAAAGTGTCACCCCTCAC3’ CAGTTCAGCATGAGGAAGAGG3’	192–206 pb
***IL1A***	rs3783553	INDEL	F-5’TGGTCCAAGTTGTGCTTATCC3’ R-5’ACAGTGGTCTCATGGTTGTCA3’	230–234 pb
***IL4***	rs79071878	VNTR	F-5’AGGGTCAGTCTGGCTACTGTGT3’ R-5’CAAATCTGTTCACCTCAACTGC3’	147/217/287 pb
***MDM2***	rs3730485	INDEL	F-5’GGAAGTTTCCTTTCTGGTAGGC3’ R-5’TTTGATGCGGTCTCATAAATTG3’	192–232 pb
***TP53***	rs17880560	INDEL	F-5’TCCATTCATAACTCAGGAACCA3’ R-5’TTAAATCCCGTAATCCTTGGTG3’	135–141 pb
***TYMS***	rs151264360	INDEL	F-5’ATCCAAACCAGAATACAGCACA3’ R-5’CTCAAATCTGAGGGAGCTGAGT3’	213–219 pb
***UCP2***	–	INDEL	F-5’CCCACACTGTCAAATGTCAACT3’ R-5’CCATGCTTTCCTTTTCTTCCT3’	119–164 pb
***UGT1A1***^a^	rs8175347	VNTR	F-5’CTCTGAAAGTGAACTCCCTGCT3’ R-5’AGAGGTTCGCCCTCTCCTAT3’	133/135/137/139 pb
***XRCC1***	rs3213239	INDEL	F-5’GAACCAGAATCCAAAAGTGACC3’ R-5’AGGGGAAGAGAGAGAAGGAGAG3’	243-247pb
***MiR-146ª***	rs2910164	SNP	C__15946974_10	–
***MIR-499***	rs3746444	SNP	C___2142612_30	–
***MiR-196a2***	rs11614913	SNP	C__31185852_10	–
***MiR-149***	rs2292832	SNP	C__11533078_1_	–

^a^Allele 5 and 6 are Del and allele 7 and 8 are Ins

### Ancestry informative markers (AIMs)

AIMs were used to adjust for population admixture, controlling population stratification and avoiding spurious associations in case-control studies. Genetic ancestry contributions of European, African, and Amerindian ancestry was investigated using a set of 61 autosomal AIMs described by Santos et al. 2010 and Ramos et al. [15, 16].

### Statistical analyses

Statistical analyses were performed using the RStudio v.1.3 software. Group comparisons for categorical variables were performed using the chi-squared test, whilst the t-Student test was used to analyze the continuous variables. Multiple logistic regression analyses were performed to estimate the odds ratio (OR) with 95% confidence intervals (CI). These analyses were adjusted for confounding variables including gender, comorbidities, smoking, genetic ancestry. A 5% significance level was used for the analyses. Deviation from Hardy-Weinberg equilibrium was assessed using chi-squared tests with Bonferroni correction. The results were analyzed comparing the genotypic distributions adopting the dominant model and they estimated values ​​of *p* corrected for multiple tests through FDR (False Discovery Rate) test.

## Results

We evaluated the association of 13 INDEL and four SNPs variants and susceptibility of tuberculosis in an Amazon region with a high degree of interethnic admixture. Demographic and clinical characteristics of individuals with tuberculosis and healthy controls were presented in Table 1. The mean age of participants was 50 ± 16.84 in the case and 52 ± 9.15 years old in controls. The differences in control and TB groups were statistically significant for gender, comorbidity, smoking, and ancestry. In the tuberculosis group, 47.7% of participants were male and 52.3% were female, in contrast with the control group, in which 17.3% were male and 82.7% were female. For the case group, 107 out of 283 cases had comorbidities, which were primarily cancer, diabetes, non-drug hepatitis, and autoimmune diseases. Smoking habits were higher in case groups (31.7%) than in the control group (20.7%).

Populational ancestry was evaluated using three reference populations: European, African, and Amerindian. The ethnic composition of the case group was 25.4%% African, 40.9% European, and 33.7% Amerindian, while in the control group composition was 21.5% African, 49.6% European, and 28.9% Amerindian. The population structure results showed a lower European contribution and a higher African and Amerindian contribution in the cases when compared to the control group.

The associations between 17 polymorphisms and tuberculosis risk were presented in Table 3. The association of genotypes was performed through logistic regression with adjustment for potential confounding factors including age, gender, comorbidity, smoking, and genetic ancestry. The polymorphisms investigated in this study were in accordance with the Hardy–Weinberg equilibrium test. Allele and genotypes of several polymorphisms studied were not associated with tuberculosis risk, except rs4646994 in the *ACE* gene.

**Table 3:** Association between groups for the 17 polymorphisms investigated

GENE	PATIENTS n (%)	CONTROLS n (%)	***p*** ^***c***^	OR (95% CI) ^**d**^
*IL1A* (rs3783553)				
Ins / Ins	64 (23.2%)	30 (20.7%)		
Ins / Del	128 (46.4%)	70 (48.3%)	0.844	0.934 (0.472–1.847)
Del / Del	84 (30.4%)	45 (31.0%)		
Ins	0.46	0.45		
Del	0.54	0.55		
TYMS (rs151264360)				
Ins / Ins	53 (19.2%)	14 (09.7%)		
Ins / Del	120 (43.5%)	66 (45.5%)	0.202	1.719 (0.78–3.950)
Del / Del	103 (37.3%)	65 (44.8%)		
Ins	0.41	0.32		
Del	0.59	0.68		
*UGT1A1* (rs8175347)				
Ins / Ins	116 (42.6%)	65 (46.1%)		
Ins / Del	115 (42.3%)	55 (39.0%)	0,132	0.978 (0.950–1.007)
Del / Del	41 (15.1%)	21 (14.9%)		
Ins	0.64	0.66		
Del	0.36	0.34		
*CYP2A1* (rs17880560)				
Ins / Ins	241 (86.3%)	120 (82.8%)		
Ins / Del	37 (13.3%)	25 (17.2%)	0.101	1.842 (0.887–3.825)
Del / Del	1 (0.40%)	0 (0%)		
Ins	0.93	0.91		
Del	0.07	0.09		
*SGSM03* (rs56228771)				
Ins / Ins	108 (39.1%)	74 (51.0%)		
Ins / Del	122 (44.2%)	57 (39.3%)	0.326	0.752 (0.427–1.327)
Del / Del	46 (16.7%)	14 (9.7%)		
Ins	0.61	0.71		
Del	0.39	0.29		
*IL4* (rs79071878)				
Ins / Ins	43 (15.6%)	21 (14.5%)		
Ins / Del	133 (48.2%)	68 (46.9%)	0.631	0.829 (0.384–1.786)
Del / Del	100 (36.2%)	56 (38.6%)		
Ins	0.4	0.38		
Del	0.6	0.62		
*MDM2* (rs3730485)				
Ins / Ins	18 (6.5%)	11 (7.6%)		
Ins / Del	122 (44.2%)	58 (40.0%)	0,174	0.457 (0.148–1.412)
Del / Del	136 (49.3%)	76 (52.4%)		
Ins	0.29	0.23		
Del	0.71	0.72		
*ACE (rs4646994)*				
Ins / Ins	17 (6.0%)	11 (7.6%)		
Ins / Del	120 (42.4%)	49 (33.8%)	**0.005**	**0.465 (0.272–0.794)**
Del / Del	146 (51.6%)	85 (58.6%)		
Ins	0.27	0.25		
Del	0.73	0.75		
*CCR5 (rs333)*				
Ins / Ins	1 (0.4%)	0 (0.0%)		
Ins / Del	18 (6.5%)	15 (10.3%)	0.413	0.680 (0.270–1.712)
Del / Del	257 (93.1%)	130 (89.7%)		
Ins	0.03	0.05		
Del	0.97	0.95		
*UCP2 (no rs)*				
Ins / Ins	131 (47.5%)	74 (51.0%)		
Ins / Del	113 (40.9%)	57 (39.3%)	0.361	0.774 (0.447–1.340)
Del / Del	32 (11.6%)	14 (9.7%)		
Ins	0.68	0.71		
Del	0.32	0.29		
*HLA-G (rs371194629)*				
Ins / Ins	77 (26.0%)	49 (33.8%)		
Ins / Del	144 (48.6%)	72 (49.6%)	0,635	0.866 (0.478–1.568)
Del / Del	75 (25.3%)	24 (16.6%)		
Ins	0.44	0.58		
Del	0.56	0.41		
*XRCC1 (rs3213239)*				
Ins / Ins	13 (7.4%)	10 (6.9%)		
Ins / Del	88 (50.0%)	56 (38.6%)	0.109	0.390 (0.123–1.234)
Del / Del	175 (42.6%)	79 (54.5%)		
Ins	0.21	0.26		
Del	0.79	0.74		
*TP53 (rs17880560)*				
Ins / Ins	196 (71.0%)	101 (69.7%)		
Ins / Del	74 (26.8%)	42 (29.0%)	0.610	1.64 (0.649–2.087)
Del / Del	6 (2.2%)	2 (1.4%)		
Ins	0.84	0.84		
Del	0.16	0.16		
*miR-146A (rs2910164)*				
CC	128 (46.6%)	67 (47.9%)		
GC	123 (44.7%)	57 (40.7%)	0.256	1.753 (0.666–4.617)
GG	24 (8.7%)	16 (11.4%)		
C	0.69	0.68		
G	0.31	0.32		
*miR-192A (rs11614913)*				
TT	23 (8.4%)	9 (6.7%)		
CT	119 (43.4%)	55 (40.7%)	0.113	1.578 (0.888–2.773)
CC	132 (48.2%)	71 (52.6%)		
T	0.3	0.27		
C	0.7	0.73		
*miR-499 (rs3746444)*				
GG	21 (7.4%)	10 (6.9%)		
GA	111 (39.2%)	55 (37.9%)	0.166	0.717 (0.447–1.149)
AA	151 (53.4%)	80 (55.2%)		
G	0.27	0.26		
A	0.73	0.74		
*miR-149 (rs2292832)*				
CC	36 (13.2%)	19 (13.2%)		
TC	115 (42.3%)	66 (45.8%)	0.206	0.639 (0.320–1.279)
TT	121 (44.5%)	59 (41.0%)		
C	0.34	0.36		
T	0.65	0.63		

^**c**^ *p*-value obtained by regression adjusted logistics for age, gender, comorbidity, smoking, and genetic ancestry, adopting the dominant model. The *p* values ​​were corrected for FDR multiple test; ^d^ *Adjusted Odds Ratio* (OR)

**Fig. 1:**
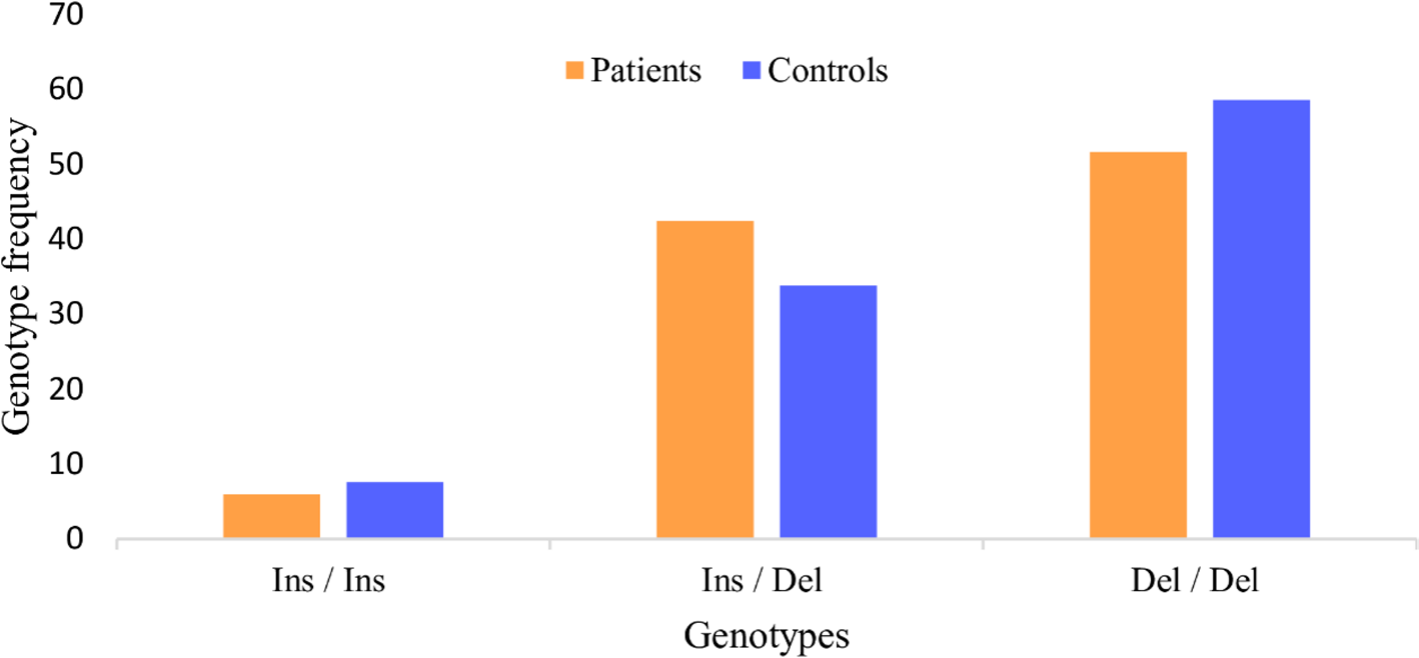
Distribuição das frequências genotípicas para o marcador rs4646994 (*ACE*) no grupo de pacientes de TB e grupo controle

Only the rs4646994 polymorphism in the *ACE* gene showed significant differences between the cases and controls. For this SNP, the Ins/Del alleles in the case group were 27% for Ins and 73% for Del, and the Ins/Del alleles in the control group were 25% for Ins and 75% for Del. The frequency of Del allele (rs4646994) was higher in control than tuberculosis group. Moreover, the homozygous Del/Del genotype for this SNP was present in 51.6% of cases and 58.6% of controls. The rare Ins/Ins genotype (rs4646994) was present in only 6% of cases and 7.6% of controls. The results showed that ACE Del/Del genotype was associated with protection to tuberculosis development, in the domain model (OR = 0.465; *p* < 0.005). Figure 1 provided a visual illustration of the differences between genotypic frequencies for ACE Ins/Del polymorphism (rs4646994).

## Discussion

Tuberculosis remains a major health problem worldwide. Several studies suggested the association of host genetic factors to TB susceptibility in various ethnic populations. The prevalence of tuberculosis disease was higher in males in this study. In the study of Hertz and Schneider (2019) and Ben et al. (2020), the tuberculosis infection was significantly higher in men than in women, a phenomenon reported in several countries [17, 18]. There is a lack of information on the role of gender in this disease. Although, there are some potential explanations why male had an increased odds of developing tuberculosis infection. Some researchers suggest the influence of sex-specific determinants of immunity, which include effects of sex steroid hormones and sex chromosome-encoded genes and miRNAs [19]. In addition, men are more exposed to factors that can compromise immunity, such as illicit drugs, smoking, and comorbidities such as diabetes and HIV [17, 20, 21].

Comorbidities and smoking are also two important risk factors widely associated with TB, with their deleterious effect especially relevant in countries with a high prevalence of the disease [22], and our work corroborates that these two variables are linked to susceptibility to the disease in the Amazonian population. It is known that patients with comorbidities are at greater risk of developing TB compared to people without comorbidities due to changes in the immune response, with HIV, diabetes, and hepatitis being the most common comorbidities in patients with TB [23, 24]. Smoking has a harmful effect on the lungs since it decreases the phosphorylation of transcriptional factors that regulate the expression of IFN-g [25], altering the immune response and also hindering the functioning of immune cells such as macrophages, monocytes, and CD4 lymphocytes [26, 27].

We also have explored the association between *ACE* polymorphisms and tuberculosis in the Amazonian Brazilian population, which is characterized by a genetic background of three parental populations (European, African, and Amerindians). Similar results from our ancestry analysis were found by Leal et al. where the Amerindian contribution was significantly higher and the European contribution was significantly lower in the Tuberculosis group in the Brazilian Amazon population [27].

One of the primary physiological roles of *ACE* is in both innate and adaptive responses by modulating macrophage and neutrophil function. In particular, macrophages that overexpress ACE are more effective against tumors and infections. Neutrophils that overexpress ACE have an increased production of superoxide, which increases their ability to kill bacteria [28]. Recent studies point to the functionality of *ACE* in promoting the maturation of myelocytic lineage cells and to improve the effective phagocytosis capacity and consequent self-limitation of Mtb infection [29, 30]. In this context, individuals with Del/Del genotype would have greater gene expression that would have a crucial role in the innate effective immune response.

We found Del/Del genotype of the *ACE* gene (rs4646994) showed significant association by setting up a protective factor for the development of tuberculosis. To our knowledge, few studies evaluated the correlation between *ACE* polymorphisms and the risk of tuberculosis. Ogarkov et al. investigated the occurrence of frequencies of *ACE* genotype in Russian individuals with tuberculosis The-Ins/Del polymorphism of the gene *ACE* was studied and the Del/Del genotype (*ACE*) was significantly higher in the group of TB patients when compared to controls [31]. However, Zhang et al. also analyze the Ins/Del polymorphism and development of tuberculosis. The results showed that the Ins/Del polymorphism studied was not associated with susceptibility to TB in the Chinese population [32]. The study by Ogarkov et al. [31] and Zhang et al. [32] found different results than our results. The reason may be differential susceptibility to TB can vary significantly between ethnic populations, due to genetic factors such as genomic ancestry, and environmental factors, such as poverty, unequal access to health, and the environment [33].

Many data indicate the association between *ACE* polymorphism, particularly the homozygote variant (Del/Del), might contribute to the risk of chronic obstructive pulmonary disease (COPD) and COPD with pulmonary hypertension among Asians [8]. Similar findings were described by Gomez et al., who found that the *ACE* Del/Del polymorphism was statistically higher in the severe COVID-19 group than in the mild disease group [6]. Furthermore, Dmitrenko et al. found that the Del/Del genotypes were associated with increased risk of pregnancy complication in gestational diabetes mellitus women, increasing its risk by 2,96 [11]. Zmorzynski et al. observed the association between Del/DEl genotype and more than 2-fold risk of multiple myeloma [34]. The Del/Del genotype was significantly more frequent in coronary heart disease (CHD) patients with type2 diabetes mellitus (T2DM) compared to each of CHD patients without T2DM and control populations [35].

Interestingly, the *ACE* polymorphism and tuberculosis protection in Amazon populations were significantly associated, however larger studies are necessary to deepen the relationship between the ACE gene and its potential protective factor for tuberculosis infection in the Amazonia population. Further studies are necessary to validate these findings for other ethnic populations. It is important, as well, to investigate other genetic markers like polymorphisms in the immune response genes in this population.

## Conclusion

In conclusion, host genetic factors may play a role in tuberculosis disease, influencing susceptibility to infection. Our case-control study suggests that the Del/Del genotype of the rs4646994 in *ACE* gene was associated with protective factors to tuberculosis. The identification of genetic variants responsible for susceptibility to tuberculosis may help develop new and improved diagnostic tools for tuberculosis infection. This genetic study can help improve TB control and the future eradication of this disease.

## Data Availability

The datasets are available in https://figshare.com/s/0af7c9a15996be56a062 or DOI 10.6084/m9.figshare.14551035.
